# Showing and giving: from incipient to conventional forms

**DOI:** 10.1098/rstb.2021.0102

**Published:** 2022-09-12

**Authors:** Gideon Salter, Malinda Carpenter

**Affiliations:** School of Psychology and Neuroscience, University of St Andrews, Scotland, UK

**Keywords:** gesture, showing, giving, development, infancy

## Abstract

Understanding humans' motivation and capacity for social interaction requires understanding communicative gestures. Gestures are one of the earliest means that infants employ to communicate with others, and showing and giving are among the earliest-emerging gestures. However, there are limited data on the processes that lead up to the emergence of conventional showing and giving gestures. This study aimed to provide such data. Twenty-five infants were assessed longitudinally at monthly intervals from 6 to 10 months of age using a variety of methods (elicitation procedures, free play observations and maternal interviews), as well as via questionnaires conducted at 11–12 months. A particular focus was on pre-conventional, *incipient gestures*, behaviours that involved some components of conventional gestures, but lacked other important components. We present observational evidence that at least some of these behaviours (observed as early as 7 months of age) were communicative and make the case for how conventional showing and giving may emerge gradually in the context of social interactions. We also discuss the influence of maternal interpretations of these early behaviours on their development. Overall, the study seeks to draw attention to the importance of understanding the cognitive, motor and interactional processes that lead to the emergence of infants’ earliest communicative gestures.

This article is part of the theme issue ‘Revisiting the human ‘interaction engine’: comparative approaches to social action coordination’.

## 1. Showing and giving: from incipient to conventional forms

Understanding the human ‘interaction engine’ [1] requires understanding the development of communicative gestures. If Levinson [1] is indeed correct that human interactions are unique, based on a capacity for cooperative and reciprocal engagement that is not dependent on language, then it is necessary to understand the kinds of behaviours that appear in some of humans' ontogenetically earliest interactions, before infants have produced their first words.

Communicative gestures are among the earliest ways in which infants can initiate joint attention and communicate with others [2,3]. Much previous work has focused on pointing gestures [3–6], and many researchers have discussed the interplay of maturation and socialization that leads to the emergence of conventional pointing gestures [7–13]. However, if we want to understand the very beginnings of gestural communication, we need to investigate other key, earlier-emerging gestures such as showing and giving [14–18]. Showing involves holding up objects so that others can see them [14], and giving involves placing and releasing an object into another's hand [19]. These gestures typically first emerge at around 9 to 10 months, typically before pointing [2,3,14], and, like pointing, they are associated with later language [18,20]. It is therefore striking that there is so little research on the development of these earlier-emerging communicative gestures.

For the emergence of showing gestures, the literature is particularly sparse. Although several studies have documented when showing emerges in its conventional form [2,3,14,15], none has explored where showing comes from developmentally. By contrast, some studies have explored the development of giving gestures. Carpendale *et al*. [21] reported diary entries in which caregivers described the giving behaviours of their children from 7.5 months to 2 years, 6 months of age. They reported some descriptions of behaviours that preceded conventional giving gestures. For example, caregivers reported infants putting food or other objects into the caregiver's mouth as an early form of giving. Other studies have highlighted the role of give-and-take interactions in giving development, using case studies [22,23], observations [24] and experiments [19,25]. For example, Xu *et al*. [19] demonstrated the influence of social experience on the development of giving in response to a request. They compared two one- to two-week interventions in which 7.5-month-old infants either engaged in give-and-take interactions with their caregiver or had experience of putting a toy into a bucket. Only the interactive intervention led to a significant increase in giving, and infants in the interactive condition gave objects significantly more than those in the bucket condition.

In understanding the developmental pathway to conventional gestures, it is important to establish the key features that mark a gesture as conventional. Broadly speaking, conventions are reliable patterns in social interaction that facilitate communication and coordination [26]. Conventional gestures are recognizable by caregivers, with a consistent behavioural form that is used regularly and predictably in communicative contexts [2]. Importantly, they are also recognizable by members of the community beyond the caregiver–infant dyad [2].

The current study explores the very beginnings of infants' early showing and giving gestures, focusing on where they come from developmentally, and the transition from pre-conventional to conventional forms. That is, conventional showing and giving do not emerge without developmental precedent, so we examined related behaviours—partial, attempted, unclear or idiosyncratic forms of the gesture—that emerged in infants before conventional showing and giving. We label these forms *incipient gestures*. Our aim was not just to document these earlier incipient forms, but also to explore how they relate to the emergence of conventional forms. We also considered whether infants intended them to be communicative, presenting evidence that some of these incipient gestures were accompanied by classic signs of intentional communication, like eye contact, and were not just random behaviours that coincidentally resembled showing and giving in some ways. Through maternal interview data, we also considered the role that caregiver interpretations might play in the development of these conventional gestures.

Data for this study were taken from a larger study on the development of joint attention and communication by Salter [27]. One aim of the larger study was to catch the very beginnings of communication; thus, we focused on 6- to 10-month-old infants. Longitudinal observations of 25 infants’ behaviours were made from experimental tasks designed to elicit communicative gestures, as well as free play interactions between mothers and infants. This provided a range of contexts for observation, while also, in the former case, providing situations in which the response of the recipient was controlled and did not involve the scaffolding typically provided by caregivers [8]. Maternal reports, taken from semi-structured interviews that included questions about the development of gestures, were also examined. Understanding what caregivers interpret as gestures, or possible gestures, is relevant for two main reasons. First, it provides insight into the kinds of behaviours that produce responses from caregivers, and thus that serve as an input into the cycles of interactive engagement that have been highlighted as a key facet of social development [8,22]. Second, caregivers have a large number of opportunities to engage with and observe their infant, in a range of social situations [28]. Thus, they are uniquely positioned to observe subtle and gradual developmental changes and infrequently-occurring behaviours [7]. Observational data were collected and interviews were conducted monthly when infants were 6–10 months old, and caregivers remotely provided further information through questionnaires at 11–12 months.

## 2. Method

### (a) Participants

Twenty-five mother–infant dyads participated (14 female infants, 11 male; 14 firstborns; all full term). This sample size was based on previous similar studies (e.g. [3,14]). Of the 23 infants whose mothers provided information about educational background, 20 had at least one parent who had completed tertiary education, and 3 had at least one parent who had completed secondary education. No further information on SES or ethnicity was collected, though all mothers were fluent English speakers (the tasks and interviews were all conducted in English). Sessions were conducted in the laboratory within a week of infants' monthly birthdays, once a month from 6 to 10 months, and only one infant missed a single session (at 10 months). Because the focus of the larger study was on the very beginnings of joint attention and communication, the laboratory sessions were only conducted from 6 to 10 months. Follow-up questionnaires were provided at 11 and 12 months to provide information about the further development of participants’ joint attention and communication.

### (b) Procedure

The overall study of which the gesture procedures were a part involved a number of different tasks (e.g. joint attention, attention following and imitation). The session started with a 6 min mother–infant free play period, followed by the experimental tasks (including the gesture tasks), followed by the maternal interview.

#### (i) Showing

Showing gestures were examined in two experimental procedures and the free play period. The two experimental procedures were conducted in random order before and after, respectively, the other tasks taking place at the session, and followed a similar format. Infants sat on a mat in front of their mother, facing the experimenter (E), who kneeled in front of them approximately 1 m away. E gave infants a toy (e.g. a rubber, plastic or soft toy) and then left the room, at which point mothers had been instructed to either swap the toy for a new toy, or just sit with infants for 10 s. This created two situations in which a showing gesture could be encouraged; one in which infants might share an object that had previously been shared with E [29], and one in which they might share an object that had not previously been shared with E [6]. E returned after the swap or after 10 s. He again kneeled 1 m in front of infants and greeted them. He then verbally expressed interest either in the toy (in the task in which it was swapped: ‘Wow!’; ‘That's cool!’; ‘Can you show me the toy?’) or in infants (in the other task: ‘Hello!’; ‘How are you?’; ‘Are you having fun?’). Each of these three utterances was repeated, with a 5 s pause between each utterance. Whether the infant had produced a showing gesture or not, E then waited a further few seconds, with a neutral but pleasant expression, before moving on to the rest of the session.

In the free play, mothers and infants were positioned facing each other, with infants either sitting independently or upright in an infant seat for the first half, and then positioned however the mother wanted in the second half (sitting independently or lying). They were given a bag containing a range of different toys (e.g. rattle, board book and rubber bath toys). Mothers were asked to interact with infants as they typically would at home.

#### (ii) Giving

Giving gestures were examined in a single experimental task. Infants sat in an infant chair opposite E, who sat on the floor, such that the infants' and E's eyes were at approximately the same level. Between them was a small table. Infants were given a small rubber toy (e.g. frog or ball). E allowed infants to explore the toy briefly, then extended his hand towards infants with a palm-up gesture, and requested the object, saying, ‘Hi [infant's name], can I have the toy, please?’ He did this up to three times, pausing for 5 s between each repetition. If infants clearly released the object into his hand, E thanked them and the task ended. If infants had not done so after the third request, E continued with the rest of the session.

Giving was not coded from the free play. We were interested in understanding infants' capabilities, and it was difficult to determine these in free play as mothers frequently intervened to take the objects before it was possible to identify whether infants would release the object themselves. It is important to note that this means that the giving gestures were elicited, not spontaneous. However, infants' successful responses indicated an understanding of the request and the ability to produce a giving gesture.

#### (iii) Maternal interviews

Each month, E asked mothers questions as part of a semi-structured interview that covered a range of topics relating to their infant's development. Two of these questions focused on gestures:

‘Does your infant produce any gestures? If so, what kind of gestures?’

‘Does your infant show you interesting objects? If so, how do they do this?’

As needed, E asked follow-up questions, such as asking about infants' giving or pointing gestures. The interviews were transcribed. When infants were 11 and 12 months old, mothers were also sent an online questionnaire that included a number of interview questions, including the two listed above.

### (c) Coding

Coding of both showing and giving followed the same conceptual structure, which distinguished conventional from incipient gestures; table 1. The electronic supplementary material contains the full coding scheme for each gesture type (sections S1 and S2) and information on inter-rater reliability (table S1).

**Table 1.** Conceptual structure of gesture coding schemes (see electronic supplementary material, sections S1 and S2 for the detailed coding scheme for each gesture type).

score	description
2	Infants produced a conventional instance of the target gesture.
1	Infants produced an attempt at the target gesture, a partial instance of the gesture, or otherwise relevant behaviour that involved components of the target gesture but that was missing one or more key components. This code includes behaviours that may possibly be considered an instance of the conventional gesture, but cannot be confidently assigned as such.
0	Infants did not produce a gesture or other relevant action.

The definitions of showing and giving were broadly in line with previous work (e.g. [14,17]), but with some additional stipulations. For conventional showing (a score of ‘2’), infants had to raise the object into the view of the adult, keep it stably in place for at least 1 s and look to both the adult's face and the object. For a score of ‘2’, the coders had to be confident that the act was produced to draw the adult's attention to the target object. For conventional giving, infants had to place the object in E's hand and release it such that the object remained in E's hand.

## 3. Results and discussion

### (a) Showing

**Figure 1.**
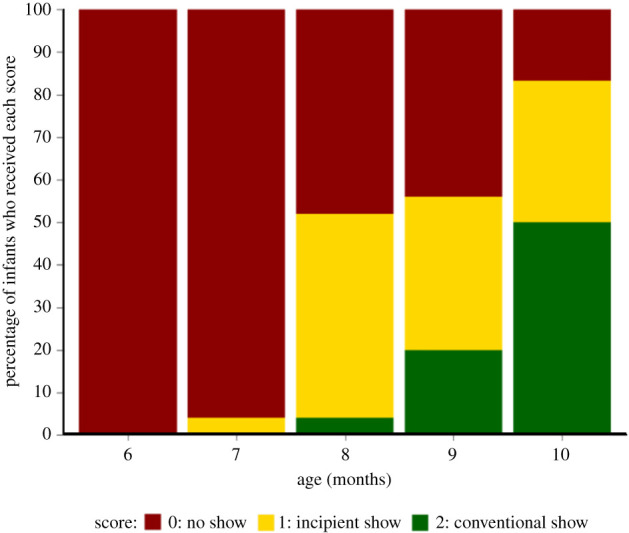
Percentages of infants who received scores of ‘0’, ‘1’ and ‘2’ as their highest score for showing gestures at each month.

Figure 1 presents the percentage of infants who received each score as their highest score for showing gestures at each month, collapsed across both showing tasks and free play. The first infant to produce a conventional showing gesture was 8 months old, in the free play. The number of infants who produced conventional showing gestures then increased steadily, with 13 out of 25 infants (52%) having produced a conventional show at least once by 10 months. Focusing on incipient gestures, the first infant to produce an incipient show was 7 months old, in the free play. Overall, 18 out of 25 infants (72%) had produced at least one incipient showing gesture by 10 months. For individual participants' scores, see electronic supplementary material, figure S1. For information on experimental versus free play scores, see electronic supplementary material, figure S2.

In all cases, the behaviours that were coded as incipient shows were behaviours that met all but one component of the criteria for conventional showing gestures. For example, infants held the toy up towards the adult's face, with gaze alternation, but did not hold it high enough, or sufficiently stably, or they held it up stably but did not look at the adult, or did not look at the object.

The key question is how to interpret these behaviours. There are at least two possible interpretations, each ascribing different capacities to the infants. The first interpretation is that these behaviours are non-communicative and are either unrelated to conventional shows, or potentially serve as interactional triggers that elicit relevant caregiver responses. The second interpretation is that these behaviours are communicative, but not yet conventional. On this reading, the infants intend to show objects, but cannot yet do so conventionally due to limited motor and/or cognitive capabilities. It is possible that both interpretations might be correct and involved in the pathway toward conventional showing gestures, just at different moments in development. It is also possible that different infants may follow different developmental paths.

Under the first interpretation, the behaviours may be coincidental, exploratory or arousal-based: infants were simply playing with the object, or got excited by the situation, and happened to move in a way that resembled a showing gesture. One could thus conclude that these behaviours are unrelated to showing gestures, or are only related to the extent that they involve similar motor capacities to those required in order to produce the conventional behaviour. On some accounts, it is only after infants have undergone relevant cognitive developments (such as means-ends understanding [2] and understanding of attention and intentions [30]) that they can start to produce intentionally communicative gestures, suggesting that behaviours that occur prior to this understanding are not relevant. However, even if these behaviours are initially non-communicative, it does not preclude them from playing a role in the developmental pathway towards showing [7,21]. Even unintentional behaviours, as long as they are show-like, could elicit a positive response from caregivers, which in turn would encourage infants to engage in further instances of these behaviours, providing a cycle of learning from which infants could gradually become aware of the effects of these actions [21].

A second way of interpreting incipient showing gestures is that they are communicative, and that infants intend to draw the adult's attention to the object; it is just that they do not yet do this in a conventional way. In these cases, infants manifest a burgeoning capacity to engage in showing that is limited because the infants have limited motor control that prevents them from producing the conventional form and/or they do not yet fully understand the optimal form of a conventional show [2]. For example, in support of this interpretation, in the current study, there were several cases in which the behaviour almost met the criteria for a conventional show, but did not, because the object was not held stably in place, e.g.:

Participant 1, 9 months: The infant raised the object to the side of her head, making eye contact with E. She brought her arm forward so the object was held briefly (less than 1 second) towards E's face, before moving the toy up and down and smiling. Whilst shaking the toy, she attended to it briefly, and twice the up and down movement was paused so the toy was only briefly held stable in front of E.

Participant 4, 9 months: After the mother commented on a book the infant was holding and looking down at, the infant looked up at the mother, smiled, laughed, and raised the book to his head height, towards his mother's face, and lowered it, all in a single arcing motion. The mother smiled and touched the book, saying, ‘Want me to do it?’, but the infant continued to hold it.

There are several features of these examples that provide support for the thesis that these are not simply coincidentally show-like movements, but rather attempts to bring the object into the adult's visual attention communicatively. In each case, there is visual attention to both the object and the adult, and a smile produced alongside the action. The motion is towards the adult's face, either with a shaking motion or continuous movement. In the second case, the mother understood the act as bringing the object into her attention (though seemingly as a request for help). In each case, it is plausible that the infants were simply limited by their motor skills; they attempted to produce an intentional show, but lacked the coordination or strength to hold the object stably towards the adult.

Other observations suggest that at least some infants might gradually incorporate objects into engagements as they transition from engaging others communicatively in a dyadic manner, to doing so triadically. Consider the following example:

Participant 2, 8 months: The infant grasped the object, without looking at it. He then looked up to his mother, smiled, and held the object up and out in front of him, at about his head height. He held the object stable for several seconds as he rocked his body back and forth, seemingly in excitement. As he did so, his mother said, ‘What's that? What's that?’ in a high-pitched tone. The infant then lowered the object.

In this case, the infant consistently looked at his mother and smiled at her, without visually attending to the object. This suggests that he was communicatively engaging with her in a dyadic manner, rather than drawing her attention to the object. However, his raising and holding stable of the object was salient, and otherwise very much resembled a conventional show; it was clearly towards the mother and held stable for several seconds. It is relevant here that his raising of the object resulted in an excited response from the mother—one that likely would not have occurred without the inclusion of the object in the act. It is possible that this behaviour is declarative in the older sense of the word initially used by Bates and colleagues [31]: the use of an object to draw attention to oneself (see also [16] for a discussion of this behaviour). Alternatively, cases such as these may represent ‘transitional forms’ between dyadic and triadic engagements, in that it is not clear that the infant was drawing the adult's attention to the object, but the act of raising the object was salient within the engagement. On this view, objects are gradually included within the engagement, as infants become increasingly aware of the role they can play in interactions, and as caregivers increasingly react to the inclusion of objects by infants [32,33].

Infants who are motivated to engage another person with an object may require experience to learn the conventional positioning of an object when showing it—a position that is optimal spatially but also, critically, with regard to eliciting a social response. For example, some infants (e.g. Participant 15, 9 months) held the object away from their body and kept it stable, with gaze alternation between object and adult, but not clearly up and towards the adult's face. However, other features that have been used to identify intentionally communicative acts, such as waiting for a response and persisting with the act when needed [2], were not always present in these types of examples, so we cannot be sure that they were intentionally communicative (though note that with cases such as Participant 2, 8 months, above, neither of these features was necessary as the mother immediately took the act to be communicative). It may be that an infant can often succeed in getting another to attend to an object with a suboptimal (e.g. not clearly directed, unstable, with no response waiting) or non-conventional show, but it is less clear and thus less likely to elicit a response than a conventional show [15]. However, it is worth mentioning that another feature commonly used to identify communicative behaviours, eye contact, was almost always observed (with just two exceptions; see electronic supplementary material, section S3), suggesting that in most cases infants were at least engaging with the adult, and potentially intentionally communicating.

A final methodological point to highlight is the influence of the properties of the object. When infants had larger objects, or objects with protruding or dangling elements, it was sometimes less clear whether they were showing the object, for example, when they held a dangling element, e.g. a leg of a soft toy, with the main body of the object hanging below (e.g. Participant 13, 10 months). Furthermore, the size and/or weight of the object may have been the source of some of the problems with stability. A unique challenge of showing gestures (compared to, for example, pointing) is that infants actively control the position of the object themselves, and thus the objects' size and shape play a role in the form of the showing gesture that is produced. Any multimodal properties the object has can also be relevant; for example, sometimes infants might share the noise made by an object, the movements it can make, or its tactile properties. A possible avenue for future research is to explore infants’ capacity to show these different properties of objects. However, if the focus of a study is solely on infants' production of conventional showing gestures, the best objects to use, in our experience, are small, compact, lightweight, easily graspable and silent.

To summarize, the majority (72%) of infants produced at least one incipient showing gesture between 7 and 10 months, that is, a behavioural sequence that included several components of a showing gesture, but lacked a key component. We have highlighted different plausible ways to interpret these gestures and have suggested ways in which they might be implicated in the developmental pathway toward the emergence of conventional shows. In particular, we have highlighted the possibility that infants might have some limited capacity to integrate objects into communicative exchanges prior to learning to produce conventional showing gestures, as well as the possibility that they may intend to show objects, but are limited by their motor capabilities or not having learnt all the features of a conventional show.

### (b) Giving

**Figure 2.**
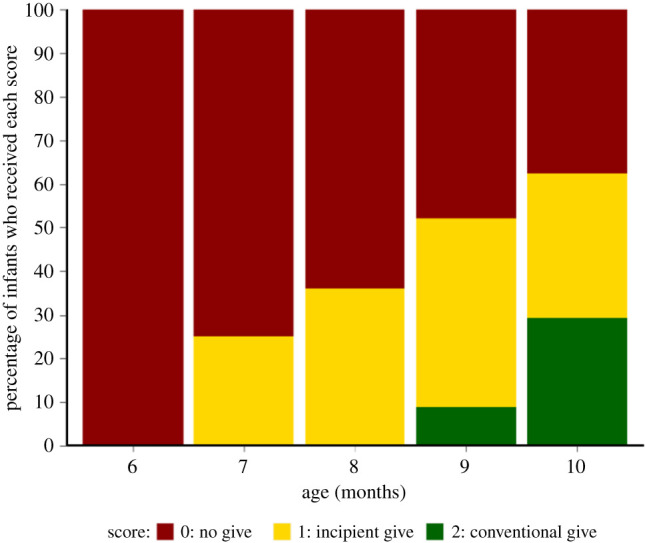
Percentages of infants who received scores of ‘0’, ‘1’ and ‘2’ as their highest score for giving gestures at each month.

Figure 2 presents the percentage of infants who received each score as their highest score for giving gestures at each month.

The first infants to produce a conventional giving gesture were 9 months old (2 out of 25; 8%). Conventional giving increased somewhat between 9 and 10 months, with 8 infants (32%) having produced a conventional give by 10 months. Focusing on incipient gestures, the first infants (6 out of 25; 24%) to produce incipient gives were 7 months old. Overall, 18 out of 25 (72%) of infants had produced an incipient giving gesture by 10 months. For individual participants’ scores, see electronic supplementary material, figure S3.

In all cases, the behaviours that were coded as incipient gives were behaviours that met all but one component of the criteria for conventional giving gestures. For example, infants placed the object on E's hand without releasing it, or repeatedly tapped it against E's hand, or released it in an uncontrolled manner, such that it made contact with E's hand but did not remain in E's hand. Since coordinated gaze to the adult's face is a potential indicator of communicative intent, it is worth noting that only 1 out of 25 infants (4%) produced a conventional giving gesture with a look to E's face at 9 months, and 1 out of 25 (4%) did so at 10 months (a different infant). Further information regarding gaze coordination with incipient giving gestures can be found in the electronic supplementary material, section S3. However, giving gestures are frequently not coordinated with looks to the adult's face; gaze is frequently on the target object and/or the adult's hand to ensure that the object is successfully placed onto the adult's hand [14,15].

As with incipient showing gestures, there are at least two possible ways to interpret these incipient giving gestures. First, it is possible that infants did not have any understanding of E's request and instead were interacting with E's hand simply as a salient focal point for exploration. Even if so, in the context of give-and-take games, these behaviours may serve as interactional triggers for caregiver responses: as infants place the object, caregivers may encourage them to release it or simply take the object [21]. This in turn facilitates the infants' transition from a passive to an active, initiating participant [23].

Second, there are alternative plausible explanations that ascribe varying degrees of social understanding to the infants. For example, after having participated in previous interactions with caregivers in which caregivers had taken the objects from infants themselves [22–24], infants might mistakenly have believed that E wanted them simply to put the object on his hand, expecting that he would take it himself once they did this. We observed that several infants at 9 and 10 months looked up at E's face, sometimes with a smile, after initially placing the object on his hand without releasing it, suggesting that the placing was what they thought they were expected to do. It might thus be the case that at least some infants engage in ‘allowing-to-take’ before actual giving.

In other cases, it seemed clear that infants were actually trying to give the object, but did not succeed because of various limitations—most commonly motor limitations that prevented them from releasing the object in a controlled manner once it was placed on E's hand. For example:

Participant 14, 9 months: E requested the object. The infant, looking at E's hand, lowered the object onto it, holding it there for a few seconds. He smiled broadly, making eye contact with E. He then released all his fingers from the object, and it fell off E's hand and onto the table. E repeated the request twice more, and at the third request, the infant again placed the object onto E's hand. This time, he closed all his fingers and squeezed the object, which caused it to fall out of his hand, bounce off E's hand, and fall onto the table.

That the infant placed the object onto E's hand and released his fingers from the object plausibly indicates some intention to give the object to E. Other possibilities for limitations are that infants may have struggled to inhibit maintaining possession of the object, or may have failed to understand that giving requires one not just to release the object but also to leave the object in the other's hand. For example, one participant (Participant 10, 10 months), having seemingly struggled for several seconds to release the object onto E's hand (including removing his fingers from the object and sliding his hand off the object), retrieved the object immediately after releasing it onto E's hand.

These observations again suggest the possibility that these gestures, and the social understanding underlying them, emerge in a gradual manner, rather than in an all-or-nothing switch from no capacity or understanding to a mature capacity and understanding. Further observations provide evidence that infants may have had an understanding of E's request, and object transfer in general, before they produced conventional giving gestures. For example, some infants dropped the object in front of them and looked up at E (e.g. Participant 5, 9 months), or threw it towards E or E's hand after making eye contact with him (e.g. Participant 2, 9 months), which again may have been with the expectation that E obtain the object. In one case (Participant 24, 10 months), the infant grasped E's hand and moved it towards the object, which was on the table. This is potentially a ‘hand-taking gesture’ [26], encouraging E to take the object himself, and in that case, the infant ultimately did give E the object conventionally by the end of the task. The reverse also occurred: an infant (Participant 3, 9 months) grabbed E's hand and moved it away from the object, which may have been evidence that she understood but rejected his request.

To summarize, the majority (72%) of infants produced at least one incipient giving gesture between 7 and 10 months, that is, a behavioural sequence that included several components of a giving gesture, but lacked a key component. We have highlighted different plausible ways to interpret these gestures. While some of these actions may have been just exploratory, in other cases, it appeared that infants had some understanding of E's request, and object transfer more generally, before they produced conventional giving gestures.

### (c) Maternal interview responses

Mothers, too, reported incipient gestures. For example, in response to E's question about whether infants produced showing gestures, there were reports of the object not being held stable (examples are edited slightly for clarity):

Participant 7, 9 months: ‘…the occasional sort of flail that looks slightly deliberate, or more like a pause in play, and also getting eye contact’.

Participant 16, 8 months: ‘I suppose sometimes when he's banging the blocks he'll stop and he'll kind of go like this [*raises a fist and shakes it*] with his hand towards you. But I don't know if he is or not’.

And non-conventional shows:

Participant 5, 10 months: ‘He'll sometimes throw it. I feel like he throws it at me’.

Participant 6, 9 months: ‘Just holds it right up to your face’.

Participant 11, 10 months: ‘Yeah… basically she'd just bring me something, like a ‘cool’ Kleenex, she'll just put it on your lap’.

Similarly, for giving there were reports of it not being controlled:

Participant 7, 10 months: '…occasionally I can get her to give me the spoon. Mostly, she drops it, but sometimes she'll actually put it in my hand'.

Participant 13, 9 months: ‘No I don't think so, no. I think she maybe tries to. But, I don't think it has been that controlled just yet’.

And reports of non-conventional gives

Participant 15, 10 months: ‘…giving somebody something else he hasn't quite mastered, but he'll kind of drop something; he'll have it, and then will drop it and look at you, and that's almost his way of saying “Hey look!”’

In the cases of non-conventional gestures, it appears that mothers often viewed these as de facto instances of showing or giving, despite them being non-conventional. This suggests that mothers may respond not only to the form of the gesture, but also when they believe the infant is demonstrating the intention to show or give an object. This intention may be inferred through infants' use of gaze alternation, and the context, and also when the relevant interactional outcome (e.g. object transfer) is achieved, regardless of the gesture's conventionality. Conventionality may then result at least in part from mothers' increasing standards regarding what they respond to [34], combined with the infant interacting with members of the wider community, who would be more likely to understand and respond to conventional rather than idiosyncratic gestural forms.

A further noteworthy feature of the mothers’ comments is that there was often ambiguity between showing and giving, though this was less common in later assessments (i.e. at 11 and 12 months of age). Previous work has highlighted that whether an infant's initial act of holding out an object resolves as a show or a give is often an outcome of a dynamically unfolding social situation [14]. Both caregivers [15,35] and researchers can find it difficult to tell whether infants intend to show or give an object. Often, mothers responded to the interview questions about showing with a response that involved the infant transferring the object to the mother (e.g. Participant 11, 10 months above). In other cases, mothers explicitly reflected on the difficulty in interpreting the infant's potential showing or giving behaviour. For example, in response to a question about showing, some mothers said:

Participant 18, 8 months: ‘I think he is. He starts doing the bashing that he does with everything some days, but then he'll sort of catch your eye and hold things. He seems to be holding things up and he knows that if he holds certain toys like that I'll take them from him and join in and play with him. So he certainly seems to, whether or not he's meaning to’.

Participant 21, 10 months: ‘With showing… I actually grab it [*mimes grabbing*] [saying,] *‘*Oh, thank you!’ I'm not sure if she actually wants me to take it, but she does hold it towards me. Not always, though’.

These examples suggest that at these ages, sometimes the specific functional outcome is less important than the engagement with the mother. It has been suggested that infants at this age may have no social intentions and are simply carried along by the responses of the caregiver (e.g. [15]). However, it may also be the case that infants are simply open to whatever way the interaction unfolds, and only later become more particular about their specific intention being understood, perhaps because they better understand the consequences of different functional outcomes [21].

## 4. Conclusion

This study has sought to provide new insights into the behaviours that precede the emergence of some of the earliest gestures human infants produce: showing and giving. It has documented and described *incipient* gestures; behaviours that involve components of a conventional gesture but that are missing key components. Exploring a range of examples from observations and maternal reports, a case was made for why these various behaviours should be considered relevant steps along the developmental pathway to conventional forms of showing and giving. Behavioural features have been identified that indicate that young infants (as young as 7 months) may have some burgeoning capacity to engage in showing or giving, or at least intend to show or give objects, even if they are not yet capable of conventional showing and giving. On this view, conventional showing and giving are a product of a series of gradual cognitive and motoric developments that take place in the context of repeated social engagements.

Future work can explore what capacity and understanding infants might possess at different stages of gesture development, and, as some researchers have already begun to do with the development of giving [19,21–25], can also more closely examine changes at the level of the caregiver–infant dyad. By examining the capacities of infants (at the level of the infant), as well as developments in patterns of shared activity at the level of the dyad, it will be possible to articulate the key cognitive, motor and interactive processes that contribute to the emergence of infants' earliest gestures. As a methodological note, it may also be beneficial to provide a more extensive period of free play, or conduct the free play in a more natural context (e.g. the infants’ homes), to increase the likelihood of observing spontaneous gestures [18]. Additionally, greater attention could be paid to potential differences between infants' behaviour in free play interactions with caregivers versus elicitation procedures.

Finally, future work can assess caregiver understanding of incipient gestures by exploring in depth how caregivers respond to possible or partial gestures by infants in live interactions. We have suggested that incipient gestures may serve as interactive ‘triggers’ for caregiver responses, and examining caregivers' verbal and behavioural responses to incipient gestures will provide further evidence about whether and, if so, how caregivers respond to incipient gestures, and the role these responses may play in the origins of showing and giving. Caregivers could also be asked explicitly about their understanding of incipient gestures in an interview context, in order to focus more directly on their justifications for interpreting incipient or non-conventional behaviours as de facto instances of showing or giving.

More generally, this study has not only joined other recent work in calling for greater attention to be paid to infants' showing and giving gestures [14–18], but has also stressed the importance of understanding the developmental pathway that leads to the emergence of conventional showing, giving and other gestures. Pursuing this question further will help contribute to an understanding of humans' early capacity and motivation for social interaction, as well as the core role played by gestures [36]. This in turn can help address the question of precisely what are the cognitive, behavioural and motivational elements that constitute the human ‘interaction engine’ [1].

## Data Availability

The data are provided in the electronic supplementary material [37].
